# Effects of 4-Hexylresorcinol on Craniofacial Growth in Rats

**DOI:** 10.3390/ijms22168935

**Published:** 2021-08-19

**Authors:** In-Song Lee, Dae-Won Kim, Ji-Hyeon Oh, Suk Keun Lee, Je-Yong Choi, Seong-Gon Kim, Tae-Woo Kim

**Affiliations:** 1 Department of Orthodontics, School of Dentistry, Seoul National University, Seoul 3080, Korea; insong.lee@gmail.com; 2 Department of Oral Biochemistry, College of Dentistry, Gangneung-Wonju National University, Gangneung 28644, Korea; kimdw@gwnu.ac.kr; 3 Department of Oral and Maxillofacial Surgery, College of Dentistry, Gangneung-Wonju National University, Gangneung 28644, Korea; haruna348@naver.com; 4 Institution of Hydrogen Magnetic Reaction Gene Regulation, Daejeon 34140, Korea; sukkeunlee@hanmail.net; 5 Department of Biochemistry and Cell Biology, Cell and Matrix Research Institute, Korea Mouse Phenotyping Center (KMPC), School of Medicine, Kyungpook National University, Daegu 41944, Korea; jechoi@knu.ac.kr

**Keywords:** 4-hexylresorcinol, histone deacetylase, mandible, testosterone

## Abstract

4-Hexylresorcinol (4HR) has been used as a food additive, however, it has been recently demonstrated as a Class I histone deacetylase inhibitor (HDACi). Unlike other HDACi, 4HR can be taken through foods. Unfortunately, some HDACi have an influence on craniofacial growth, therefore, the purpose of this study was to evaluate the effects of 4HR on craniofacial growth. Saos-2 cells (osteoblast-like cells) were used for the evaluation of HDACi and its associated activities after 4HR administration. For the evaluation of craniofacial growth, 12.8 mg/kg of 4HR was administered weekly to 4 week old rats (male: 10, female: 10) for 12 weeks. Ten rats were used for untreated control (males: 5, females: 5). Body weight was recorded every week. Serum and head samples were collected at 12 weeks after initial administration. Craniofacial growth was evaluated by micro-computerized tomography. Serum was used for ELISA (testosterone and estrogen) and immunoprecipitation high-performance liquid chromatography (IP-HPLC). The administration of 4HR (1–100 μM) showed significant HDACi activity (*p* < 0.05). Body weight was significantly different in male rats (*p* < 0.05), and mandibular size was significantly smaller in 4HR-treated male rats with reduced testosterone levels. However, the mandibular size was significantly higher in 4HR treated female rats with increased growth hormone levels. In conclusion, 4HR had HDACi activity in Saos-2 cells. The administration of 4HR on growing rats showed different responses in body weight and mandibular size between sexes.

## 1. Introduction

Growth and development are largely controlled by genetic regulation. However, twins who share identical genomes develop remarkable differences in their genomic distribution over time [1]. This means that both genetic and environmental regulations affect gene expression and its activity, and epigenetics could be a major explanation for this phenomenon. Epigenetics means structural chromatin changes that do not involve alterations in the DNA sequence. This includes chromatin remodeling, DNA methylation, histone modification, and micro-RNAs. Of the mechanisms of epigenetic regulations, histone protein modification is the mechanism that induces gene expression and suppression by post-translational modification via acetylation, methylation, and phosphorylation in the histone N-terminal region of the nucleosome [2].

Histone deacetylases (HDACs) are enzymes that remove acetyl groups from histones and suppress gene expression by allowing for histones to bind more tightly with DNA [3]. They can perform as erasers of epigenetic marks on chromatin, counterbalanced by histone or lysine acetyltransferases (HAT/KAT), which add acetyl groups to lysines and allow for gene expression [3]. In humans, there are 18 HDAC genes that are categorized into four families (Classes I–IV) on the basis of their subcellular location, structure, and function [3]. Classes I (HDACs 1–3 and 8), IIa (HDACs 4, 5, 7, and 9), IIb (HDACs 6 and 10), and IV (HDAC11) are zinc-dependent HDACs. Class III comprises the structurally and mechanistically distinct NAD+-dependent enzyme [4].

Bone development takes place via two main processes during the early stages of embryonic development: intramembranous and endochondral bone formation. Once bone is developed, it is remodeled throughout life by the tight regulation of bone resorption and new bone formation. These whole processes require harmonized orchestration between the expression of specific key genes with transcription factors and cofactors [5]. HDACs contribute to these different steps of bone development by the deacetylation of transcription factors [6]. From studies using knockout mice, HDAC1-null embryos showed severe defects in head formation and died before endochondral ossification began [7]. HDAC2 mutant mice were reported to develop reduced body size [8]. HDAC4-deficient mice have premature mineralization of skeletal elements that prevent longitudinal growth [9]. HDAC8 knockout mice resulted in impairment of skull development [10]. These results indicate that HDACs extensively and intimately affect skeletal development and bone maintenance.

Histone deacetylase inhibitors (HDACi) are small compounds with a moiety that can integrate into zinc-containing catalytic sites of Class I and II HDACs with a capping group and linker between them. They can be classified as short-chain fatty acids (e.g., valproate and sodium butyrate), hydroxamic acids (depsipeptide, FR901228), cyclic peptides (MS-275), enzamide hydroxamic acids (SAHA, trichostatin A (TSA)), epoxyketones (trapoxin), and hybrid molecules (CHAP31 and CHAP50) according to their basic structure [11]. HDACi can upregulate gene expression by inhibiting the enzyme activity of HDAC and is used as a treatment for various diseases such as cancer, osteoporosis, arthritis, epilepsy, and mood disorder [4]. Valproic acid, sodium butyrate, and TSA are pan-HDACi that can suppress different classes of HDAC [11]. On the other hand, romidespin and entinostat (MS-275) are Class I selective HDACi [11].

Many of the studies about the effects of HDACi on skeletal development were conducted on individual bone cells and indicated the promising possibility of enhancing osteogenesis and decreasing osteoclast activity [12,13,14,15]. However, several clinical studies reported that the long-term administration of valproate decreased bone mineral density in children and adults [16,17,18], causing increased fracture risk [19]. These conflicts between in vitro and in vivo studies require further research to find the exact mechanism of how HDACi affects skeletal bone development.

4-Hexylresorcinol (4HR) is a synthetic resorcinolic lipid that has been used as an anti-parasitic and antiseptic agent since the 1920s [20]. It is also used as a food additive, as a potent inhibitor of tyrosinase, and has been tested for toxicity for a long time [21]. As 4HR is reported to have antimicrobial, anticancer, and anti-inflammatory effects, diverse attempts are in progress to utilize 4HR in different medical and biological fields [22]. Interestingly, 4HR played a role as an HDACi in a previous study [23]. Valproate was developed as an antiepileptic drug [16,17,18], but 4HR is used as a food additive [21]. Accordingly, the chance for exposure would be higher in 4HR compared to other HDACi. If HDACi influences skeletal growth, 4HR administration could change the morphology of the facial skeleton and body weight. Therefore, we examine (1) the effects of 4HR on HDAC activity in Saos-2 cells (osteoblast-like cells), and (2) the effects of 4HR on the mandibular skeletal development and the body weight in rats.

## 2. Results

### 2.1. 4HR Administration Inhibits Class I HDAC Activity and Increases the Acetylation Level of Cellular Proteins

**Figure 1:**
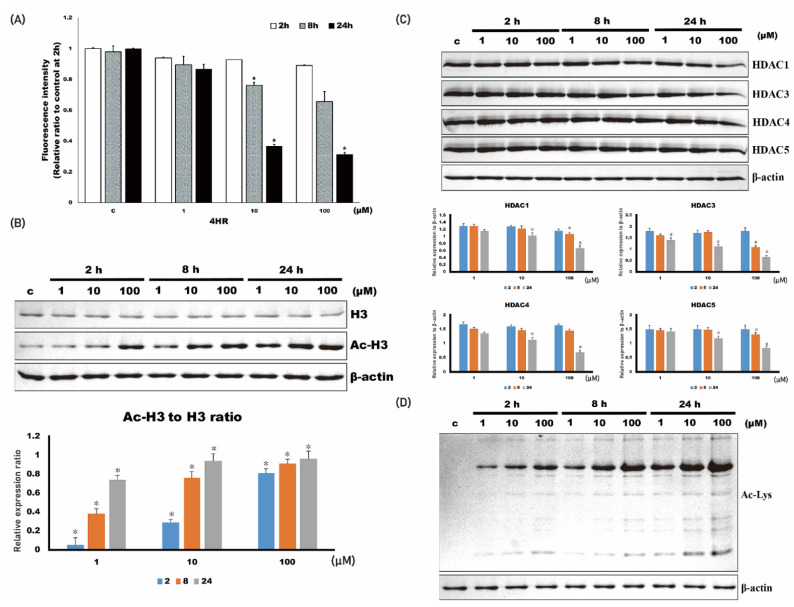
4HR administration and histone deacetylase activity in Saos-2 cells. The administration of 4HR decreased histone deacetylase activity and the expression level of HDAC1, 3, 4, and 5. (**A**) Histone deacetylase activity decreased upon 4HR administration. When compared to untreated control, 10 and 100 μM 4HR group showed significantly lower values at 8 and 24 h after administration (* *p* < 0.05). (**B**) The level of acetylated histone 3 (Ac-H3) increased by 4HR administration. When Ac-H3 to H3 ratio of untreated control was compared, 4HR administered groups showed significantly higher values (* *p* < 0.05). (**C**) Expression level of HDAC1, 3, 4, and 5 decreased by 4HR administration. When compared to untreated control, 10 and 100 μM 4HR group showed significantly lower values at 8 and 24 h after administration (* *p* < 0.05). (**D**) Protein acetylation level generally increased by 4HR administration, as shown in whole cellular lysates.

The activity of HDAC was decreased by the administration of 4HR (1–100 μM; Figure 1A). When HDAC activity was compared among groups, its activity in Saos-2 cells treated with 4HR (1 μM–100 μM) decreased significantly at 8 and 24 h (*p* < 0.001 and 0.009, respectively). In the post hoc test, 10 and 100 μM 4HR treatments resulted in significantly lower values compared with those of the untreated control group at 8 h (*p* = 0.003 and <0.001, respectively). When observed at 24 h after 4HR treatments, 100 μM 4HR treatments resulted in significantly lower values compared with those of the untreated control group (*p* = 0.009).

The expression level of HDAC1, 3, 4, and 5 was decreased by 4HR administration (Figure 1B). When the relative expression level of each HDAC to β-actin was compared among groups, the expression level in Saos-2 cells treated with 4HR (1–100 μM) significantly decreased (*p* < 0.001). In the post hoc test, 100 μM 4HR treatments resulted in a significantly lower expression level of HDAC1 compared with that in the untreated control group at 8 h (*p* = 0.038). When observed at 24 h after 4HR treatments, 10 and 100 μM 4HR treatments resulted in the significantly lower expression level of HDAC1 compared with the untreated control group (*p* = 0.006 and <0.001, respectively); 10 and 100 μM 4HR treatments resulted in the significantly lower expression level of HDAC3 compared with the untreated control group at 8 h (*p* < 0.001 and =0.018, respectively). When observed at 24 h after 4HR treatments, 10 and 100 μM 4HR treatments resulted in a significantly lower expression level of HDAC3 compared with in the untreated control group (*p* < 0.001); 10 and 100 μM 4HR treatments resulted in a significantly lower expression level of HDAC4 compared with in the untreated control group at 24 h (*p* = 0.007 and <0.001, respectively); 10 and 100 μM 4HR treatments resulted in significantly lower expression level of HDAC5 compared with in the untreated control group at 24 h (*p* = 0.001 and <0.001, respectively).

The acetylated histone 3 (Ac-H3) was increased by 4HR administration (1–100 μM; Figure 1C). When the relative expression ratio of Ac-H3 to H3 was compared among groups, the ratio in Saos-2 cells treated with 4HR (1–100 μM) increased significantly (*p* < 0.001). In the post hoc test, 4HR treatments (1–100 μM) resulted in a significantly higher ratio of AC-H3 to H3 compared with that in the untreated control group (*p* = 0.003 for 1 μM at 2 h and *p* < 0.001 for the other groups). The protein acetylation level for whole-cell lysates demonstrated that 4HR administration increased protein acetylation level (Figure 1D). Mitochondria are the main cellular power factories for the production of ATP—a cellular energy source. Oxygen consumption was decreased by 4HR administration (Supplementary Figure S1). Thereafter, the production of ATP was decreased by 4HR administration in Saos-2 cells (Supplementary Figure S2). Mitochondrial membrane potential (MMP) was increased by 20% after 100 μM 4HR administration (Supplementary Figure S3). The administration of 4HR decreased cytochrome c levels at 100 μM, but not other concentrations in the mitochondrial fraction (Supplementary Figure S4). Interestingly, 1 and 10 μM 4HR administration slightly increased the cytochrome c level of mitochondria without statistical significance (*p* > 0.05). Decreased cytochrome c at 100 μM of 4HR was not detected in the cytoplasmic fraction. 

### 2.2. HR Administration Decreased Serum Testosterone Level and Mandibular Size in Male Rats

**Figure 2:**
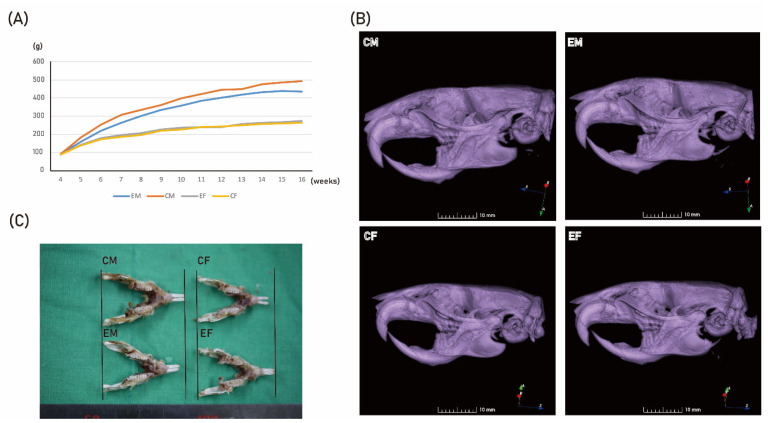
Animal study results. The administration of 4HR decreased body-weight and mandibular size in the EM group, but increased mandibular size in the EF group. (**A**) Body-weight changes in each group. Compared to the control male (CM) group, the experimental male (EM) group showed smaller body weight. However, it was almost the same between the control female (CF) and experimental female (EF) groups at all observation points. (**B**) Three-dimensional reconstructed images of micro-computerized tomogram. EM group had a smaller facial skeleton compared to that of the CM group. The CF group, on the other hand, had a smaller facial skeleton than that of the EF group. (**C**). When the mandibular size was compared among groups, mandibles from the CM group were larger than those from the EM group. However, females showed the opposite trend.

In this study, there was no significant difference in body weight between the control female (CF) and experimental female (EF) groups (*p* > 0.05). However, there was a significant difference in body weight between the control male (CM) and experimental male (EM) groups (*p* = 0.001). The body weight of CM at 16 weeks was 492.5 ± 10.5 g, and that of EM was 435.2 ± 7.5 g (Figure 2A). The size of the facial skeleton was slightly smaller in the EM group compared to that in CM group (Figure 2B). When mandible size was compared, that of the EM group was slightly smaller than that of the CM group (Figure 2C).

Mandibular size was generally smaller in the EM group compared to that in the CM group (Table 1). The EM group had significantly smaller total mandibular length II and III, corpus length I–IV, and bigonial mandibular width compared to those in the CM group (*p* < 0.05). This trend was the opposite in female rats. The measurements for the mandibular size was generally larger in EF group compared to in the CF group (Table 1). The EF group had significantly smaller total mandibular length I–III, corpus length I, and ramus height I compared to those in the CF group (*p* < 0.05).

The serum testosterone levels of the CM and EM groups were 20.38 ± 6.90 and 7.03 ± 2.10 ng/mL, respectively. The difference between groups was statistically significant (*p* = 0.036). However, those of the CF and EF groups were 0.84 ± 0.36 and 0.43 ± 0.17 ng/mL, respectively (*p* > 0.05). All groups showed similar serum 17β estradiol level values, and there was no statistically significant difference (*p* > 0.05).

In immunoprecipitation high-performance liquid chromatography (IP-HPLC), 4HR-treated male rats showed significantly decreased levels of parathyroid hormone (PTH, by 23.8%), progesterone (7.3%), testosterone (16.9%), osteopontin (12.6%), and osteoprotegerin (OPG, 7.2%) compared to the untreated male rats, while 4HR-treated female rats showed increased levels of growth hormone (GH, 12.9%), growth hormone-releasing hormone (GHRH, 5.8%), insulin (7.9%), osteocalcin (4.5%), and OPG (4%) compared to untreated female rats (*p* < 0.05). 

## 3. Discussion

**Figure 3:**
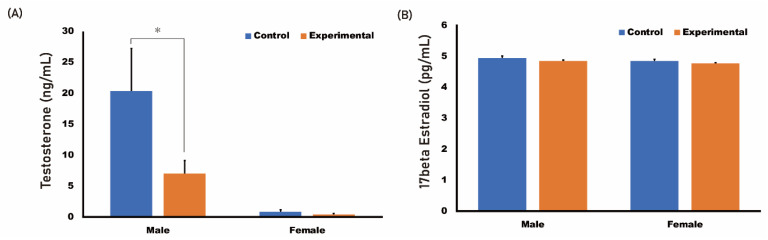
Serum level of sex hormones. Generally, the serum testosterone level was decreased by 4HR administration. (**A**) Serum testosterone level showed a significant difference between male 4HR treated and untreated rats (* *p* < 0.05). However, there was no difference in the serum testosterone level of female rats. Though the female groups also showed a similar trend, but the difference between groups was statistically insignificant. (**B**) Serum 17β estradiol level did not show any difference between groups (*p* > 0.05).

In this study, 4HR showed HDACi activity on Saos-2 cells (Figure 1A), and 4HR administration decreased HDAC activity in a time- and dose-dependent manner in Saos-2 cells. Accordingly, 4HR administration increased the acetylated protein levels in Saos-2 cells (Figure 1B,D). Subcutaneous injection of 4HR on growing rats showed reduced body weight only in males (Figure 2A). The serum level of testosterone was decreased in male 4HR-administered rats (Figure 3A). Accordingly, mandibular size was decreased in male 4HR-administered rats (Table 1 and Figure 2C). However, there was no significant body-weight difference among female rats (*p* > 0.05). Contrary to male rats, mandibular size was increased in female 4HR-administered rats (Table 1 and Figure 2C). To the best of our knowledge, this is the first report about the effect of 4HR administration on facial-morphology changes in growing animals.

The 4HR is used as a food additive because of its antibrowning effect [21], and it is suspected to be pseudo-estrogen [24]. However, 4HR administration in the ovariectomized rat model did not show an estrogenic effect [25]. In this study, the serum level of estrogen was not significantly different between the EF and CF groups (*p* > 0.05; Figure 3B), and 4HR showed HDACi activity in HUVECs [23]. Like other HDACi, 4HR has anticancer [26] and anti-inflammatory [27] effects. In this study, the HDACi activity of 4HR was confirmed in Saos-2 cells (Figure 1).

**Figure 4:**
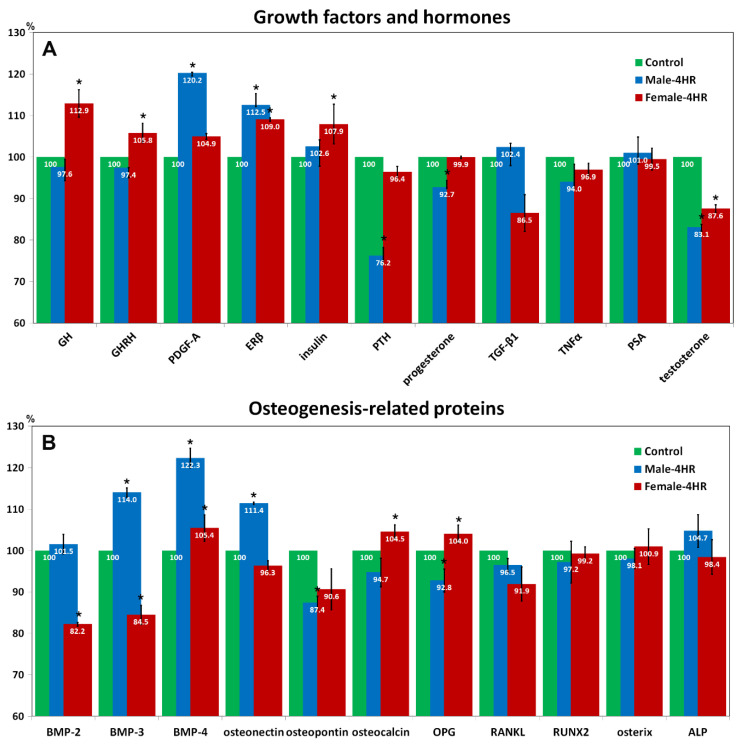
IP-HPLC analysis. Expression of growth factors, hormones, and osteogenesis-related proteins in the blood plasma of rats. Expression profiles of (**A**) growth factors and hormones, and (**B**) osteogenesis-related proteins. (* *p* < 0.05 when compared to the control). 4HR-treated male rats showed significantly decreased levels of parathyroid hormone (PTH, 23.8%), progesterone (7.3%), testosterone (16.9%), osteopontin (12.6%), and osteoprotegerin (OPG, 7.2%) compared to the untreated male rats, while 4HR-treated female rats showed increased levels of growth hormone (GH, 12.9%), growth hormone-releasing hormone (GHRH, 5.8%), insulin (7.9%), osteocalcin (4.5%), and OPG (4%) compared to untreated female rats (PDGF-A: platelet-derived growth factor-A, Erβ: estrogen receptor-β, TGF-β1: trans-forming growth factor-β1, TNFα: tumor necrosis factor-α, PSA: prostate-specific antigen, BMP: bone morphogenic protein, RANKL: Receptor activator of NF-κB, RUNX2: Runt-related transcription factor 2, ALP: alkaline phosphatase).

In this study, 4HR administration did not show a significant difference in the female body weight (Figure 2). However, 4HR administration to the male rats delayed the increase of body weight, approximately a week behind (Figure 2A). The male-only difference was an interesting phenomenon. The serum level of testosterone was significantly lower in EM group compared to that in CM group (*p* < 0.05; Figure 3A). This result was confirmed again in IP-HPLC assay (Figure 4A). However, the serum level of testosterone was highly variant in male laboratory rats and it was 2–48 nm/L [28]. Though the EM group showed a significantly smaller value than that of the CM group, the value of EM group was also in the physiological range (Figure 3). Thus, body weight and the mandibular-size difference between groups might be more important results (Figure 2). The serum level of testosterone in the pre-pubertal stage is important in determining mandibular size. When newborn mice receive an orchiectomy, mandibular growth is significantly reduced [29]. The effect of ovariectomy on mandibular size is less significant compared to that of orchiectomy [29]. However, the effect of orchiectomy on the mandibular alveolar bone is shown as a delayed pattern in 3-month-old rats compared to that of ovariectomy [30]. Ovariectomy increased the body weight of 3-month-old female rats, but orchiectomy did not change the body weight of 3-month-old male rats [30]. In a later study of the same group, orchiectomy delayed body-weight gain in 3-month-old male rats [31]. In this study, mandibular bone mineral density was significantly different at 150 days postoperatively by orchiectomy, and not by ovariectomy [31]. Aromatase is an enzyme that converts testosterone into estrogen. The single-nucleotide polymorphism of aromatase results in a significant difference in mandibular growth only in human boys and not girls [32]. In this study, a decrease in testosterone levels upon 4HR administration resulted in a decrease in mandibular size (Figure 2 and Figure 3). Contrary to our experimental design, the administration of anabolic steroids in five-week-old female rats showed the opposite phenomena of an increase in body weight and skull length [33].

Few papers have been published on the relationship between HDACi and testosterone level. Valproic acid was developed as an antiepileptic drug, and it shows HDACi activity [11]. The administration of valproic acid into male mice decreased serum testosterone levels [34]. On the other hand, valproic acid administration on cultured porcine ovarian follicular cells increased testosterone secretion [35]. Therefore, the effect of valproic acid administration may be different depending on the administration method and animal model [34]. A single injection of valproic acid on newborn mice increased the acetylation level of H3 [35]. In this study, 4HR administration increased H3 acetylation in Saos-2 cells. Valproic acid treatment reduced sex differences of the bed nucleus of the stria terminalis [36]. Reduced steroid hormone by valproic acid administration is mainly mediated by mitochondrial stress [37]. Interestingly, 4HR induced mitochondrial stress in Saos-2 cells (Supplementary Figures S1–S4). There might be a similar mechanism between 4HR and valproic acid in steroid-hormone-associated cellular response, but it should be clarified in a further study. Sodium butyrate is also an HDACi [11]. The effect of testosterone on *C. elegans* behavior can be eliminated by sodium butyrate administration [38]. Acute sodium butyrate administration increases H3 acetylation [38].

Patients having hypoparathyroidism showed decreased mandibular cortical thickness [39]. Sanjad-Sakati syndrome is associated with hypoparathyroidism and micrognathia [40]. The EM group showed small mandibles with a reduced PTH level (Figure 2 and Figure 4A). Though PTH regulates HDAC4 in UMR 106-01 cells [41], the exact mechanism between HDACi and PTH expression is yet to be clarified. Noggin is a BMP-4-inhibitory protein. Noggin polymorphism is also associated with a small mandible [42]. The serum level of BMP-4 was increased in the EM group (Figure 4).

Contrary to male rats, female rats showed increased mandibular size after 4HR administration (Table 1). The EF group showed elevated GH and GHRH levels compared to those of the CF group (Figure 4). GH deficiency showed small mandibles [43]. Rats that were small for gestational age showed reduced body weight with increased HDAC and could be treated by exogenous GH administration [44]. The administration of trichostatin A, which is an HDACi, increased GH-mRNA expression level in pituitary cells [45]. However, the EM group did not show elevated expression of GH and GHRH (Figure 4). Reduced testosterone level might have had a much stronger effect on skeletal development than that of other hormones in the EM group. Testosterone level in the EF group was also lower than that in the CF group (Figure 3A and Figure 4A). Because the baseline level of testosterone was too low in female rats, this difference induced by 4HR administration might not have had a strong effect on skeletal development. Instead of the testosterone effect, the elevated expression of GH and GHRH in the EF group might have induced mandibular growth (Table 1). However, the mechanism in sexual difference to 4HR administration is yet to be clarified in detail.

## 4. Materials and Methods

### 4.1. Saos-2 Cell Culture

Saos-2 cells (Korean cell line bank, Seoul, Korea) were grown in culture dishes. The medium was RPMI 1640 (ThermoFisher Scientific, Waltham, MA, USA) supplemented with fetal bovine serum and antibiotics. Cells were cultured in a humidified CO_2_ incubator at 37 °C. 

### 4.2. Western Blot and HDAC Inhibitory Assay

When Saos-2 cells were grown, approximately 70% confluent, they were treated with 1, 10, and 100 µM 4HR for 2, 8, or 24 h; control cells were treated with 0.1% dimethyl sulfoxide in culture medium. Cultured cells were harvested with 0.01% trypsin and 1 mM EDTA. Cellular lysis was performed with a protein lysis buffer (PRO-PREPTM, iNtRON Biotechnology INC, Sungnam, Korea). Collected lysates were used for the Western blotting of HDAC1, HDAC3, HDAC4, HDAC5, Ac-lys, H3, and Ac-H3. Antibodies for HDACs and Ac-lys were purchased from SantaCruz Biotechnology (Santa Cruz, CA, USA). The quantification of the proteins was performed as previously described [22,23]. 

HDAC enzyme activity after 4HR administration was assessed by a commercially available kit (CAT: ab156064, Abcam, Cambridge, UK). Saos-2 cells received 1, 10, and 100 μM of 4HR, and cellular lysates were collected after 2, 8, and 24 h. The subsequent procedure was in accordance with the manufacturer’s protocol. The activity of HDAC1, 2, 3, and 8 (Class I HDAC) was measured with this kit according to the product datasheet. HDAC assay buffer and substrate were added to the reaction wells. Inhibitor and developer were placed into wells and thoroughly mixed. The prepared samples were added to each well and incubated for 20 min at room temperature. Then, the stop solution was added and incubated for 10 min at room temperature. Fluorescence intensity was measured with a plate reader.

### 4.3. ATP, Mitochondrial Membrane Potential (MMP), and Oxygen-Consumption Assay

ATP was detected using a luminescent ATP detection assay kit (CAT: ab113849, Abcam). To assess the effect of 4HR administration on ATP levels, 1, 10, and 100 μM 4HR were applied, and cellular lysates were collected after 8 and 24 h. The subsequent procedure was in accordance with the manufacturer’s protocol. Briefly, the ATP standard was added into standard wells, and the medium was added into the control wells. The detergent solution was added and incubated for 5 min. Then, the substrate solution was added and incubated for 5 min. The plate was stored in a dark room for 10 min. Luminescence was measured using a plate reader.

MMP was measured using a commercially available kit (CAT: MAK159, Sigma-Aldrich). Cells were prepared on plates. JC-10 Dye Loading Solution was prepared by adding 50 μL of 100× JC-10 to 5 mL of Assay Buffer A. Cells were treated with 10 μL of 10× test compounds to induce apoptosis. In parallel, we set up negative (vehicle only) and 4HR-treated samples. Cells were incubated for 24 h. JC-10 Dye Loading Solution (50 μL) was added to each well. Cells were protected from light and incubated in a 5% CO_2_ and 37 °C incubator for 30 min. Assay Buffer B was added at 50 mL to each well. Fluorescence intensity was measured at 490 and 540 nm. The ratio of red/green intensity was used to determine MMP.

The oxygen-consumption assay was performed using an oxygen-consumption assay kit (CAT:ab197243, Abcam). Briefly, Saos-2 cells (4.0 × 10^4^ cells/well) were plated on 96-well cell-culture plates and incubated overnight. After removing the media from all wells, they were replaced with 150 μL of fresh culture media. Reconstituted extracellular O_2_ consumption reagent (10 μL) was added to each sample well. For the blank control well, 10 μL of fresh culture medium was added. Each well was sealed by adding 100 μL of prewarmed high-sensitivity mineral oil. For the measurement, the prepared plate was inserted into a fluorescence plate reader preset to the measurement temperature (37 °C).

### 4.4. Immunoprecipitation High-Performance Liquid Chromatography (IP-HPLC)

Arterial blood plasma was obtained from 4-week-old rats treated with 4HR (12.8 mg/kg) for 12 weeks separately, and each 20 μL plasma was immunoprecipitated using antisera of GH, GHRH, PDGF-A, ERβ, insulin, PTH, progesterone, TGF-β1, TNFα, PSA, testosterone, BMP-2, BMP-3, BMP-4, osteonectin, osteopontin, osteocalcin, OPG, RANKL, RUNX2, osterix, and ALP, followed by HPLC analysis. Proportional data (%) were plotted on a line graph and a star plot. The expression of housekeeping proteins, i.e., β-actin, α-tubulin, and glyceraldehyde 3-phosphate dehydrogenase (GAPDH) was compared to that of the nonresponsive control (≤5%) at 8, 16, or 24 h after 4HR treatment.

### 4.5. Animal Experiments

The animal experiment was approved by the Institutional Animal Care and Use Committee of Gangneung-Wonju National University (GWNU-2021-2-1). In total, 30 rats were used in this study. All the rats were 4 weeks old and came from the same grandmother and grandfather. Fifteen males and fifteen females were separately caged. The initial weight of males at the time of arrival was 87–90 g, and that of females was 85–86 g. The dosage of 4HR was 12.8 mg/kg weekly, injected subcutaneously. Body weight was measured weekly, and 4HR injection was performed for 12 weeks. When rats were 16 weeks old, all were humanely sacrificed. Rats were anesthetized with enfluorane, and an additional injection was performed. Whole blood was collected from the heart and stored in a heparinized tube. The collected tube was centrifuged, and the supernatant serum was collected for further analysis. After sampling the blood, animals were killed by paraformaldehyde injection. Tissue samples were collected from the masseter muscle, kidneys, liver, pituitary gland, salivary gland, and ovaries. Head samples were sent for mCT analysis.

### 4.6. Microcomputerized Tomography (mCT)

The rat heads were used for mCT analysis. Images were taken using a μCT50 (Scanco Medical, Brüttisellen, Switzerland) at the Center for Scientific Instruments, Gangneung-Wonju National University (Gangneung, Korea). The source voltage was set as 90 kVp. An aluminum filter was used, and the image pixel size was 9.04 μm. Scanned images were reconstructed using the 3D Slicer 4.11 software (free software, available online: www.slicer.org accessed on 7 August 2021).

**Figure 5:**
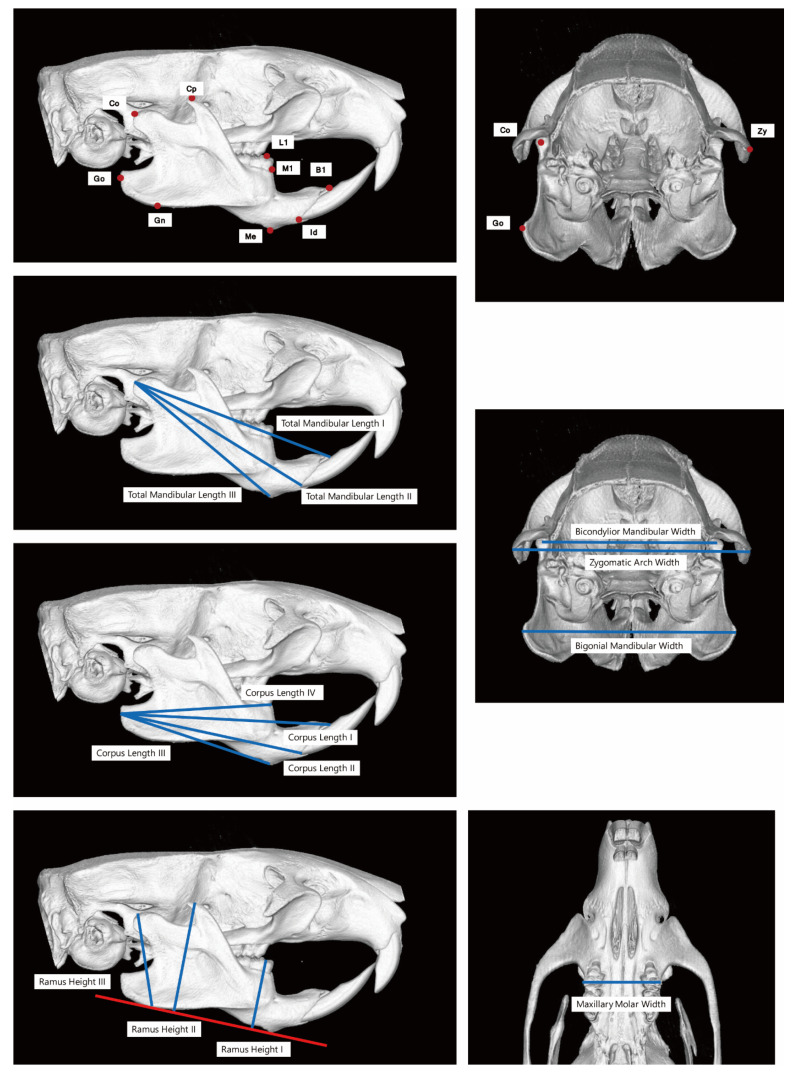
Measurements in mCT images. The definition of each landmark are shown in Table 2.

Linear measurements were conducted on the scanned images. The definition of landmarks is shown in Table 2. The definition of measurements is shown in Figure 5. Briefly, total mandibular length I was the distance from Co to B1. Total mandibular length II was the distance from Co to Id. Total mandibular length III was the distance from Co to Me. Corpus length I–IV was Go–B1, Go–Id, Go–Me, and Go–M1, respectively. Ramus height I–III was L1–L1’, Cp–Cp’, and Co–Gn, respectively. Bicondylar mandibular width was from Co to Co, maxillary molar width was from L2 to L2, and zygomatic width was from Zy to Zy.

## 5. Conclusions

In conclusion, the administration of 4HR on Saos-2 cells decreased Class I HDAC activity and the expression level of HDAC4 and HDAC5, which are Class IIa HDAC. Subcutaneous administration of 4HR on growing rats exhibited different results between sexes. Male 4HR-administered rats showed reduced body weight with small mandibles, but female 4HR-administered rats showed no significant body weight difference with larger mandibles.

Though the administration of 4HR has an influence on the growth of the facial skeleton, a lot of detailed mechanisms are yet to be clarified. As 4HR has been used as a food additive, its potential influence on the growth of facial skeleton in children should be studied.

## Figures and Tables

**Table 1:** mCT measurements. The mandibular size was generally smaller in the EM group compared to that in the CM group. However, the measurements for the mandibular size were larger in the EF group compared to in the CF group. The unit for each measurement was mm.

	Experimental Males (EM)		Control Males (CM)		Experimental Females (EF)		Control Females (CF)	
	Mean	SE	Mean	SE	Mean	SE	Mean	SE
Total mandibular length I	27.39	0.16	27.86	0.18	25.70 *	0.18	24.94	0.09
Total mandibular length II	25.69 *	0.19	26.35	0.17	24.18 *	0.17	23.61	0.11
Total mandibular length III	23.63 *	0.17	24.19	0.13	21.92 *	0.15	21.41	0.10
Corpus length I	27.81 *	0.16	28.54	0.20	26.47 *	0.21	25.73	0.16
Corpus length II	24.42 *	0.19	25.43	0.22	23.37	0.19	22.91	0.18
Corpus length III	21.26 *	0.15	22.20	0.16	20.09	0.17	19.88	0.24
Corpus length IV	20.06 *	0.14	20.80	0.17	18.92	0.17	18.52	0.14
Ramus height I	9.48	0.06	9.39	0.08	8.59 *	0.07	8.19	0.09
Ramus height II	14.75	0.08	15.13	0.13	13.27	0.10	12.98	0.09
Ramus height III	12.41	0.11	12.74	0.09	11.56	0.07	11.40	0.08
Bicondylior mandibular width	19.70	0.16	19.80	0.10	18.00	0.33	18.04	0.09
Bigonial mandibular width	21.75 *	0.21	22.34	0.14	18.84	0.41	18.51	0.37
Maxillary molar width	9.58	0.05	9.61	0.06	9.28	0.16	9.30	0.08
Zygomatic width	25.27	0.12	25.53	0.25	23.66	0.21	23.10	0.19

* *p* < 0.05.

**Table 2:** The landmarks in mCT. The anatomic location of each landmark was shown in Figure 5.

Condylion	Co	Most posterosuperior point of condylar process
Gonion	Go	Most posterior point of angular process of mandible
Gnathion	Gn	Point on most inferior contour of angular process of mandible
Menton	Me	Point on most inferior contour of lower border of mandible, adjacent to incisors
Infradentale	Id	Most inferior point of the marginal alveolar bone of the lower central incisor
	M1	Point on intersection between the mandibular alveolar bone and mesial surface of first molar
	B1	Point on intersection between lingual surface of lower incisor and anteriormost part of lingual alveolar bone
	L1	Point on mesial occlusal fossa of lower first molar
	L1’	Crossing point on Me–Gn perpendicular to Me–Gn from L1
	L2	Most external point on buccal surface of lower first molar
Coronoid Process	Cp	Point on most superior contour of coronoid process
	Cp’	Crossing point on Me–Gn perpendicular to Me–Gn from Cp
Zygion	Zy	Most external point of the zygomatic arch

## Data Availability

Raw data can be provided by the corresponding author on request.

## Supplementary Materials

The following are available online at https://www.mdpi.com/article/10.3390/ijms22168935/s1.
