# Supplementary material for: Effects of 4-Hexylresorcinol on Craniofacial Growth in Rats
Source: Int J Mol Sci. 2021 Aug 19;22(16):8935. doi: 10.3390/ijms22168935 (PMC8396282; doi:10.3390/ijms22168935)

Effects of 4-Hexylresorcinol on Craniofacial Growth in Rats

In-Song Lee [1], Dae-Won Kim [2], Ji-Hyeon Oh [3], Suk Keun Lee [4], Je-Yong Choi [5], Seong-Gon Kim [3,*] and Tae-Woo Kim [1,*]

[1] Department of Orthodontics, School of Dentistry, Seoul National University, Seoul 3080, Korea; insong.lee@gmail.com (I.-S.L.)
[2] Department of Oral Biochemistry, College of Dentistry, Gangneung-Wonju National University, Gangneung 28644, Korea; kimdw@gwnu.ac.kr
[3] Department of Oral and Maxillofacial Surgery, College of Dentistry, Gangneung-Wonju National University, Gangneung 28644, Korea; haruna348@naver.com (J.-H.O.)
[4] Institution of Hydrogen Magnetic Reaction Gene Regulation, Daejeon 34140, Korea; sukkeunlee@hanmail.net
[5] Department of Biochemistry and Cell Biology, Cell and Matrix Research Institute, Korea Mouse Phenotyping Center (KMPC), School of Medicine, Kyungpook National University, Daegu 41944, Korea; jechoi@knu.ac.kr
* Correspondence: kimsg@gwnu.ac.kr (S.-G.K.); taewoo@snu.ac.kr (T.-W.K.); Tel.: +82-33-640-2468 (S.-G.K.); +82-2-2072-2678 (T.-W.K.)

Supplementary Figure 1. Extracellular oxygen consumption assay.

The administration of 4-hexylresorcinol (4HR) decreased extracellular oxygen consumption in Saos-2 cells. The relative oxygen consumption level compared to untreated control was 97.1 ± 1.8, 96.7 ± 1.5, and 95.4 ± 1.7 % for 1, 10, and 100 μM, respectively. Accordingly, 100 μM 4HR administration decreased 4.6% of oxygen consumption in Saos-2 cells (*p < 0.05*).

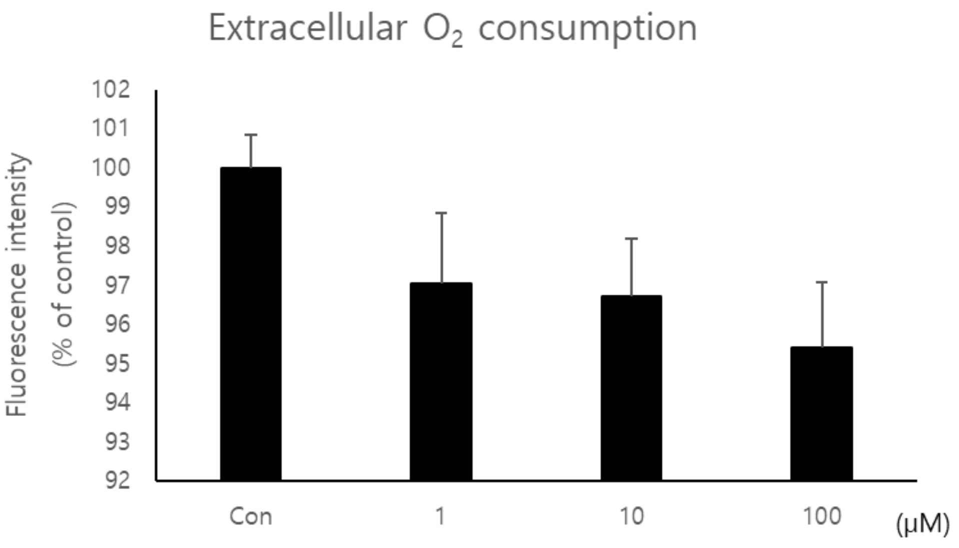

Supplementary Figure 2. ATP level assay.

The administration of 4-hexylresorcinol (4HR) decreased ATP level in Saos-2 cells. The ATP level was 54350.5 ± 4826.5, 53766.5 ± 4696.5, 51653.0 ± 3286.0 and 47338.5 ± 5362.5 RLU for untreated control, 1, 10, and 100 μM at 24 h after treatment, respectively.

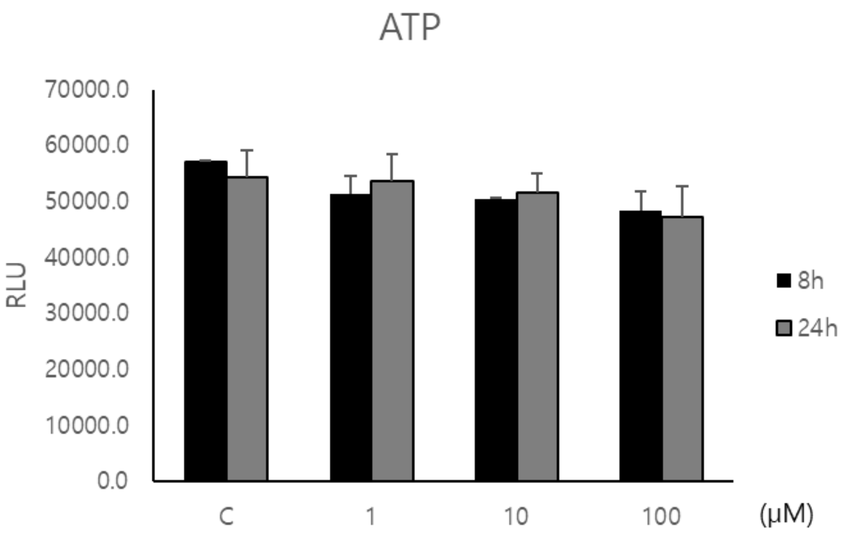

Supplementary Figure 3. Mitochondrial membrane potential assay.

The administration of 4-hexylresorcinol (4HR) increased MMP in Saos-2 cells. The relative MMP level was 103.0 ± 5.22, 108.9 ± 2.31, and 109.1 ± 2.9 % for 1, 10, and 100 μM at 24 h after treatment, respectively. Accordingly, 10 and 100 μM 4HR administration increased MMP significantly in Saos-2 cells ($p < 0.05$).

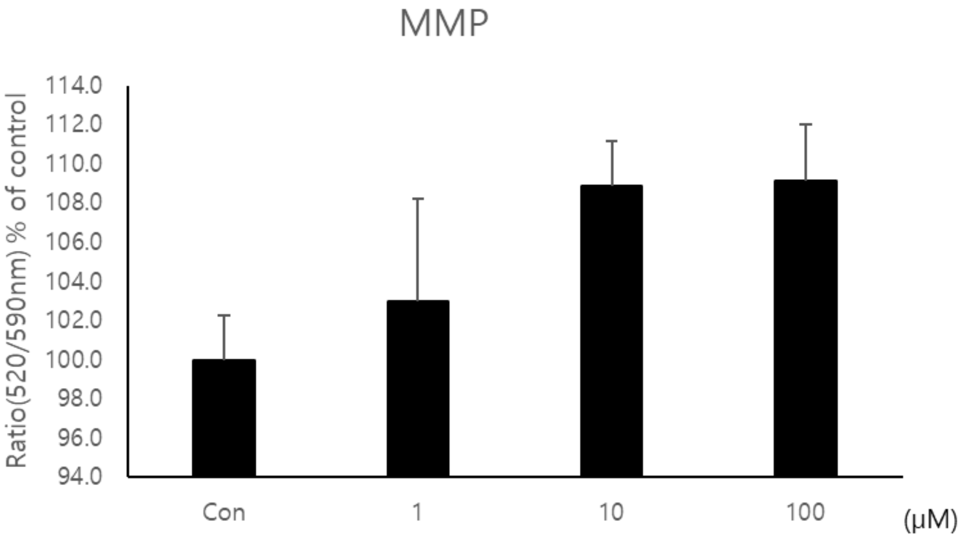

Supplementary Figure 4. Cytochrome c assay for cytoplasmic and mitochondrial fraction.

As demonstrated by Western blot analysis using cytochrome c apoptosis ICC antibody kit (Abcam, CAT#: ab110417), the administration of 4HR decreased cytochrome c levels at 100 µM, but not other concentrations in the mitochondrial fraction. Interestingly, 1 and 10 µM 4HR administration slightly increased cytochrome c level of mitochondria without statistical significance (*p > 0.05*). The decreased cytochrome c at 100 µM of 4HR was not detected in the cytoplasmic fraction.

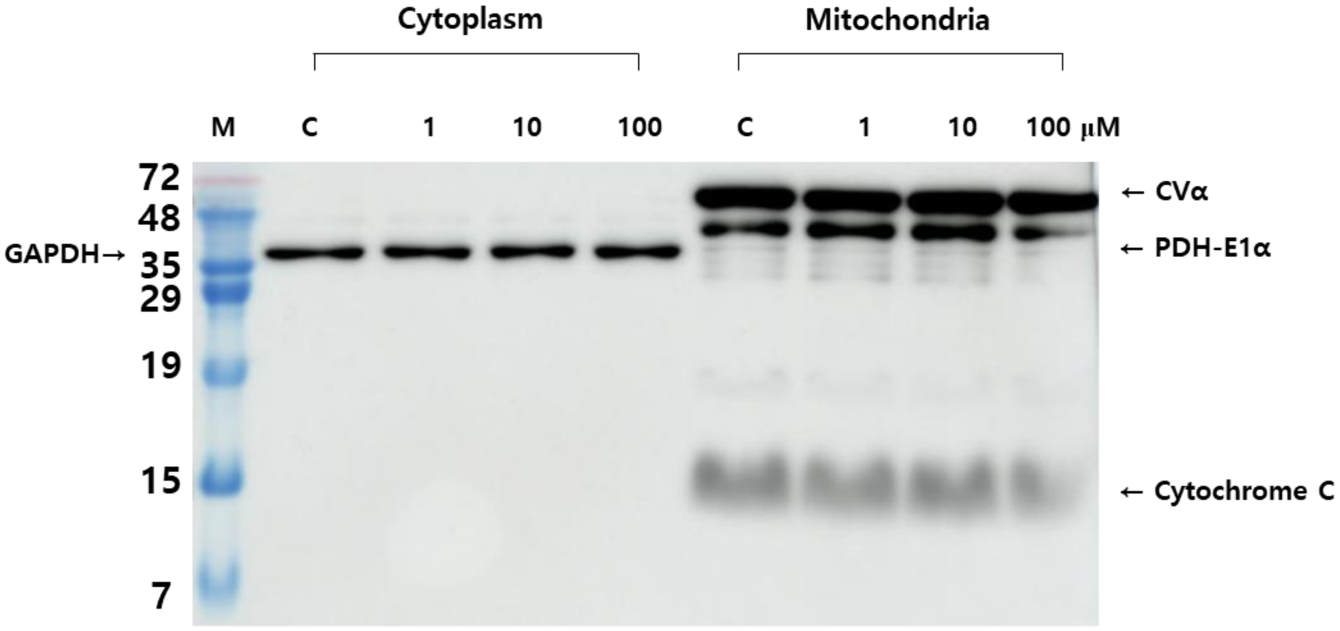

Supplement: Supplementary file 1 [file ijms-22-08935-s001.zip › ijms-1352666-supplementary.pdf]
